# Zwitterionic Surfactant-Based Silica Nanofluid for Enhanced Oil Recovery: Oil Displacement Behavior and Mechanisms

**DOI:** 10.3390/molecules31142448

**Published:** 2026-07-13

**Authors:** Zhenfeng Ma, Mingwei Zhao, Haoran Xue, Zhihao Zhang, Ying Li, Huan Zhang, Xinjie Xu, Ziyi Wang

**Affiliations:** 1 State Key Laboratory of Deep Oil and Gas, China University of Petroleum (East China), Qingdao 266580, China; b24020023@s.upc.edu.cn (Z.M.); 2402030210@s.upc.edu.cn (H.X.); b23020015@s.upc.edu.cn (Z.Z.); b25020017@s.upc.edu.cn (H.Z.); s23020029@s.upc.edu.cn (X.X.); bz23020036@s.upc.edu.cn (Z.W.); 2 Shandong Key Laboratory of Oil and Gas Field Chemistry, China University of Petroleum (East China), Qingdao 266580, China; 3 Department of Chemical and Materials Engineering, University of Alberta, Edmonton, AB T6G 1H9, Canada; ying47@ualberta.ca

**Keywords:** low-permeability reservoirs, silica nanofluid, zwitterionic surfactant, oil displacement mechanisms

## Abstract

Nanomaterials have shown great potential for enhanced oil recovery in low-permeability reservoirs because of their excellent interfacial regulation capability. However, their stability and interfacial activity are challenged under extreme reservoir conditions. In this work, a silica nanofluid composed of SiO_2_ nanoparticles and the zwitterionic surfactant lauramidopropyl hydroxy sulfobetaine (LHSB) was prepared, and its interfacial regulation characteristics and enhanced oil recovery mechanisms were systematically investigated. The results show that the introduction of LHSB effectively improves the dispersion stability of silica nanoparticles, and the prepared nanofluid maintains good stability at temperatures up to 120 °C and salinities up to 6 wt%. The silica nanofluid exhibits excellent interfacial activity and wettability alteration capability, reducing the oil–water interfacial tension to 0.57 mN/m and decreasing the static water contact angle on the rock surface from 112° to 37°. Meanwhile, the nanofluid significantly reduces flow resistance in porous media, achieving a flow resistance reduction rate of 20%. Oil displacement experiments indicate that injection volume, injection slug number, and shut-in time all have significant effects on oil recovery performance. The optimal operating condition was determined to be 1 PV single-slug injection followed by a 12 h shut-in period, under which the oil recovery during subsequent waterflooding reached 27%. We expect that this study will provide valuable insights into nanofluid formulation design, support the efficient development of low-permeability reservoirs, and offer a theoretical basis and technical guidance for field applications.

## 1. Introduction

With the continuous development of conventional medium- and high-permeability reservoirs, low-permeability reservoirs have become increasingly important for ensuring energy supply [1,2]. However, low-permeability reservoirs are typically characterized by small pore throats, low permeability, strong heterogeneity, and high fluid flow resistance [3]. In addition, due to their complex pore structure, strong capillary trapping effect, and the generally oil-wet state of rock surfaces, injected fluids have difficulty effectively entering fine pore throats and sufficiently contacting residual oil, resulting in high injection pressure, limited swept volume, and low oil recovery during conventional waterflooding [4,5,6]. These challenges become more pronounced under complex reservoir conditions, such as high temperature and high salinity, where interfacial properties and fluid flow behavior are further affected, making the efficient development of low-permeability reservoirs even more challenging [7,8,9].

To address the problems of low oil displacement recovery and limited sweep efficiency in low-permeability reservoirs, extensive studies have been carried out worldwide, leading to the development of various chemical flooding technologies, including polymer flooding, surfactant flooding, alkali flooding, foam flooding, and compound flooding [10,11,12,13]. Polymer flooding mainly improves sweep efficiency by increasing the viscosity of the displacing fluid and improving the mobility ratio [14,15]. Surfactant flooding mainly enhances microscopic oil displacement efficiency by reducing oil–water interfacial tension and altering reservoir wettability [16,17]. Alkali flooding and compound flooding improve oil recovery through the combined effects of multiple mechanisms [18,19]. In recent years, nanomaterials have attracted increasing attention in enhanced oil recovery because of their small particle size, large specific surface area, strong interfacial activity, and easy surface functionalization [20,21,22]. Nanoparticles can adsorb onto oil–water interfaces and rock surfaces, thereby reducing interfacial tension and changing reservoir wettability. In addition, the transport of nanoparticles in porous media can generate structural disjoining pressure, regulate local flow behavior, and reduce flow resistance [23,24,25,26]. These effects help improve both sweep efficiency and oil displacement recovery. Compared with conventional chemical agents, nanomaterials exhibit multiple synergistic effects and strong functional tunability, showing broad application potential for enhanced oil recovery in low-permeability reservoirs.

Although nanomaterials have shown great potential for enhanced oil recovery, they still face several critical challenges under extreme reservoir conditions, such as high temperature and high salinity. These challenges include poor dispersion stability, particle aggregation and sedimentation, and reduced interfacial activity [27,28,29]. Under high-temperature conditions, the Brownian motion of nanoparticles becomes stronger, which increases collision frequency between particles and promotes aggregation [30,31]. Under high-salinity conditions, large amounts of electrolyte ions compress the double layer on the particle surface. This weakens the electrostatic repulsion between nanoparticles and further aggravates particle aggregation, resulting in reduced system stability [32,33]. In recent years, surfactant-based nanofluids have attracted increasing attention because of their excellent thermal stability, salt resistance, and interfacial properties. Many studies have confirmed that surfactants are effective in improving the dispersion stability of nanomaterials [34,35]. Among them, zwitterionic surfactants contain both cationic and anionic functional groups. They exhibit strong temperature and salt resistance, excellent interfacial activity, and good environmental compatibility. In addition, they can maintain stable interfacial adsorption behavior under complex reservoir conditions and improve nanoparticle dispersion stability through the combined effects of electrostatic interaction and steric hindrance. Zhang et al. [36] prepared nanofluids by dispersing silica nanoparticles and five different types of surfactants in deionized water. Through interfacial tension and contact angle measurements, they revealed the oil displacement mechanisms of different nanofluids. Zhong et al. [37] prepared a highly stable active nanofluid by mixing zwitterionic hydroxypropyl sulfobetaine surfactant with silica nanoparticles. At a surfactant-to-nanoparticle concentration ratio of 0.5, the spontaneous imbibition recovery of the nanofluid was 2.61 times higher than that of API brine, 1.29 times higher than that of API brine-based nanofluid, and 0.37 times higher than that of the surfactant alone. Although many studies have reported zwitterionic surfactant-based nanofluids, relatively few studies have focused on their oil displacement behavior and mechanisms.

In this work, a novel silica nanofluid was prepared using silica nanoparticles and the zwitterionic surfactant LHSB. Its dispersion stability, thermal and salinity resistance, and interfacial regulation properties were systematically investigated. In addition, its flow resistance reduction and oil displacement performances were further evaluated. Based on these results, the effects of key operating parameters, including nanofluid injection volume, injection slug number, and shut-in time, were systematically optimized to determine the optimal operating conditions. Combined with interfacial property characterization and oil displacement results, the synergistic mechanisms responsible for enhanced oil recovery by the nanofluid were further revealed. The results of this study can provide a theoretical basis for the design of functional nanofluid formulations and technical support for enhanced oil recovery applications in low-permeability reservoirs.

## 2. Results and Discussion

### 2.1. Characterization of the Silica Nanofluid

**Figure 1:**
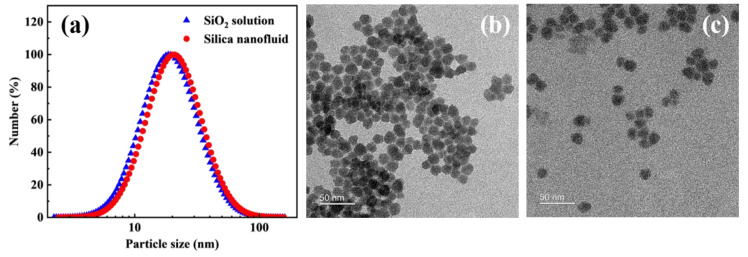
(**a**) Particle size distributions of 0.1 wt% SiO_2_ solution and silica nanofluid. TEM images of 0.1 wt% SiO_2_ solution (**b**) and silica nanofluid (**c**).

The dispersion stability of nanofluids plays a critical role in enhanced oil recovery applications. Figure 1 shows the particle size distribution and microstructural characteristics of 0.1 wt% SiO_2_ solution and silica nanofluid. As shown in Figure 1a, both systems exhibit narrow unimodal particle size distributions, mainly centered around 20 nm, indicating good particle size uniformity of SiO_2_ nanoparticles. Compared with the SiO_2_ solution, the particle size of the silica nanofluid increases slightly after the addition of LHSB. This is mainly because surfactant molecules adsorb onto the silica surface and form an adsorption layer, which increases the hydrodynamic diameter of the particles. The zeta potentials of the SiO_2_ solution and silica nanofluid are −32 mV and −40 mV, respectively, indicating good dispersion stability in both systems.

Combined with the TEM images shown in Figure 1b,c, it can be observed that a certain degree of particle aggregation exists in the SiO_2_ solution. In contrast, the particles in the silica nanofluid are more uniformly dispersed, and large aggregates are significantly reduced after the addition of LHSB. This result indicates that LHSB effectively improves the dispersion stability of SiO_2_ nanoparticles. This enhancement is mainly attributed to the adsorption of LHSB on the particle surface, which suppresses interparticle aggregation through the synergistic effects of steric hindrance and electrostatic repulsion. As a result, particle aggregation is suppressed, which improves nanoparticle dispersion in the aqueous phase and benefits subsequent interfacial regulation and oil displacement performance.

### 2.2. Thermal and Salinity Stability of the Silica Nanofluid

**Figure 2:**
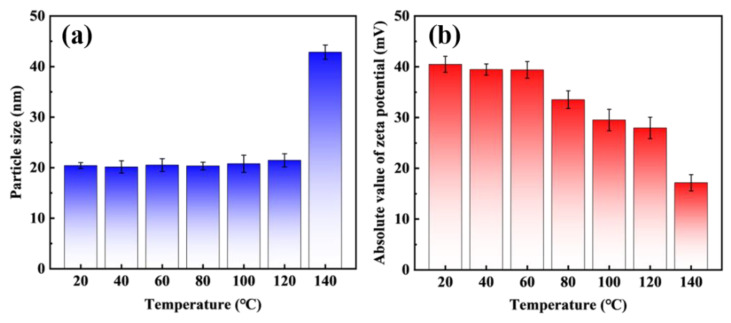
Particle size (**a**) and absolute value of zeta potential (**b**) of silica nanofluid at different temperatures.

Nanofluids tend to aggregate under high-temperature and high-salinity conditions, which may impair their oil displacement performance. Therefore, it is necessary to clarify the applicable temperature and salinity limits of silica nanofluid. As shown in Figure 2a, the particle size remains nearly constant at around 20 nm over the temperature range of 20–120 °C, with only slight fluctuations, indicating good thermal stability of the nanofluid. When the temperature increases to 140 °C, the particle size increases significantly to 43 nm, suggesting that the collision frequency between nanoparticles increases under high-temperature conditions, leading to partial particle aggregation. As shown in Figure 2b, the absolute value of zeta potential gradually decreases with increasing temperature, from 40 mV to 17 mV. According to the literature [38], nanofluids with an absolute value of zeta potential greater than 20 mV are considered to be in a stable dispersion state. Therefore, based on the combined variation in particle size and zeta potential, the silica nanofluid maintains good dispersion stability at temperatures below 120 °C, indicating its suitability for application in high-temperature reservoir environments.

**Figure 3:**
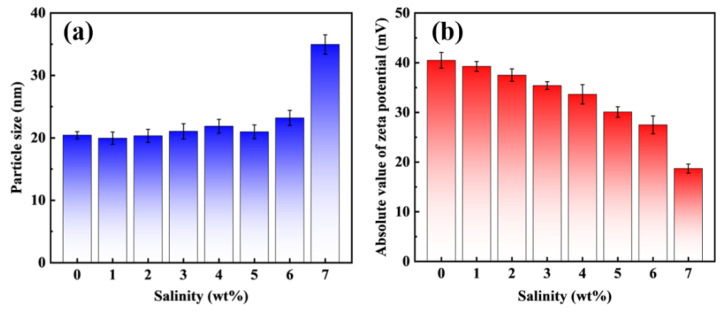
Particle size (**a**) and absolute value of zeta potential (**b**) of silica nanofluid at different salinities.

As shown in Figure 3a, the particle size remains nearly constant at around 20 nm within the salinity range of 0–6 wt%, indicating that the nanofluid maintains good dispersion stability under low-salinity conditions. When the salinity increases to 7 wt%, the particle size increases significantly to 35 nm, suggesting enhanced particle aggregation under high-salinity conditions, which reduces the dispersion stability of the system. As shown in Figure 3b, the absolute value of zeta potential continuously decreases with increasing salinity, from 40 mV to 19 mV, indicating a gradual weakening of the electrostatic repulsion between particles. This is mainly because the increase in ionic concentration compresses the double layer on the particle surface, thereby weakening interparticle electrostatic repulsion and promoting nanoparticle aggregation [39,40]. Based on the combined variation in particle size and zeta potential, the silica nanofluid maintains good dispersion stability at salinities below 6 wt%. This favorable salinity resistance enables its potential application in high-salinity reservoir environments and provides stability assurance for enhanced oil recovery under complex reservoir conditions.

### 2.3. Interfacial Tension and Contact Angle

**Figure 4:**
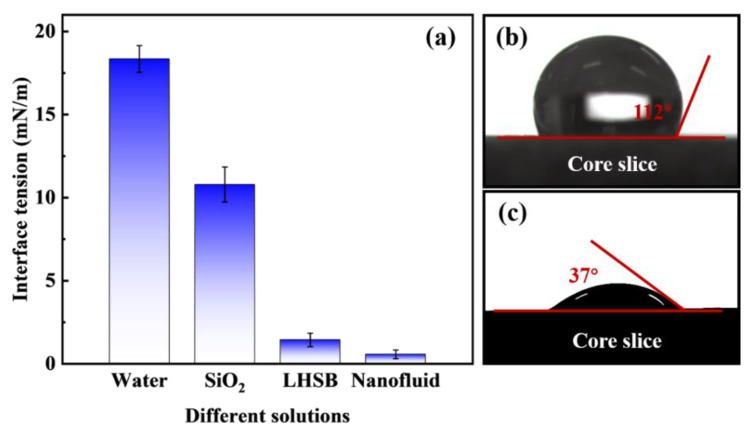
(**a**) Interfacial tensions between different solutions and simulated oil. Static water contact angles on the core surface before (**b**) and after (**c**) treatment with silica nanofluid.

The interfacial tensions between different solutions and simulated oil are shown in Figure 4a. The interfacial tension between simulated formation water and simulated oil is 18 mN/m. After the addition of 0.1 wt% SiO_2_, the interfacial tension decreases to 11 mN/m, indicating that SiO_2_ particles possess a certain adsorption capacity at the oil–water interface and can reduce the interfacial free energy, thereby lowering the oil–water interfacial tension. The interfacial tension between 0.1 wt% LHSB solution and simulated oil is 1.4 mN/m, indicating that zwitterionic surfactant molecules can rapidly migrate to and adsorb at the oil–water interface, effectively reducing the interfacial tension. For the silica nanofluid, the interfacial tension is further reduced to 0.6 mN/m, suggesting a significant synergistic effect between SiO_2_ particles and LHSB in reducing interfacial tension. This enhancement is mainly attributed to the formation of stable surface-active nanoparticles after the adsorption of LHSB onto the SiO_2_ surface. These nanoparticles not only enhance adsorption at the oil–water interface but also improve the compactness and stability of the interfacial film, thereby further reducing interfacial free energy. This is beneficial for reducing capillary force and lowering the flow resistance of crude oil.

Figure 4b,c show the variation in static water contact angle on the core surface before and after treatment with silica nanofluid. The initial water contact angle on the core surface is 112°, indicating a strongly oil-wet surface. Under this condition, the spreading ability of the water phase on the rock surface is weak, which is unfavorable for effective wetting of the pore surface by the displacing fluid. After treatment with silica nanofluid, the water contact angle decreases significantly to 37°, indicating that the rock surface is transformed from a strongly oil-wet state to a distinctly water-wet state. This result demonstrates the excellent wettability alteration capability of the nanofluid. This improvement is mainly attributed to the synergistic action of silica nanoparticles and adsorbed LHSB molecules on the rock surface. On the one hand, they can competitively adsorb onto the rock surface and displace the originally adsorbed heavy oil film, thereby reducing the coverage of the oil film on the mineral surface. On the other hand, they can form an adsorption layer enriched with hydrophilic groups on the rock surface, significantly enhancing surface hydrophilicity and altering the wettability state of the rock. The improvement in wettability helps reduce crude oil adhesion to the pore wall, thereby enhancing oil displacement efficiency [41,42].

### 2.4. Flow Resistance Reduction Rate

**Figure 5:**
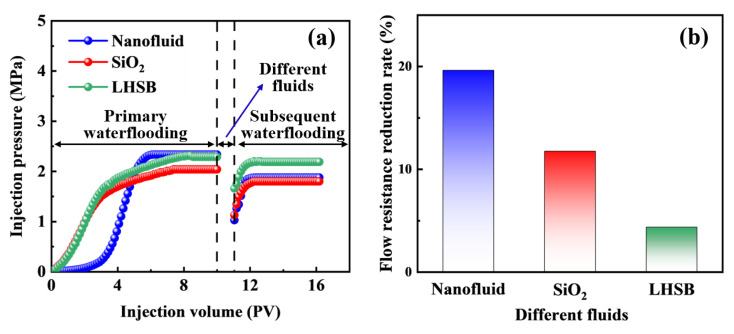
(**a**) Variation in injection pressure with injection volume during the flow resistance reduction rate tests for different systems. (**b**) Flow resistance reduction rate of different systems.

The variation in injection pressure during the flow resistance reduction rate test, together with the corresponding flow resistance reduction rate of different systems, is shown in Figure 5. During the primary waterflooding stage, the injection pressure of the three core samples gradually increased with continuous injection of simulated formation water and eventually reached stable values. After the injection of 1 PV of 0.1 wt% SiO_2_ solution, 0.1 wt% LHSB solution, and silica nanofluid, respectively, subsequent waterflooding was carried out. Compared with the primary waterflooding stage, the injection pressure of all three core samples decreased to different extents. The flow resistance reduction rates of silica nanofluid, SiO_2_ solution, and LHSB solution were 20%, 12%, and 4%, respectively. Compared with the individual SiO_2_ solution and LHSB solution, silica nanofluid exhibited a more pronounced flow resistance reduction effect, indicating a significant synergistic effect between SiO_2_ particles and LHSB. On the one hand, nanoparticles can adsorb onto the pore-throat walls, reduce surface roughness, and weaken the frictional resistance between the fluid and pore walls [43]. On the other hand, LHSB can significantly reduce oil–water interfacial tension and improve rock wettability, thereby lowering the flow resistance of fluids within pore channels.

### 2.5. Oil Displacement Behavior

#### 2.5.1. Different Systems

**Figure 6:**
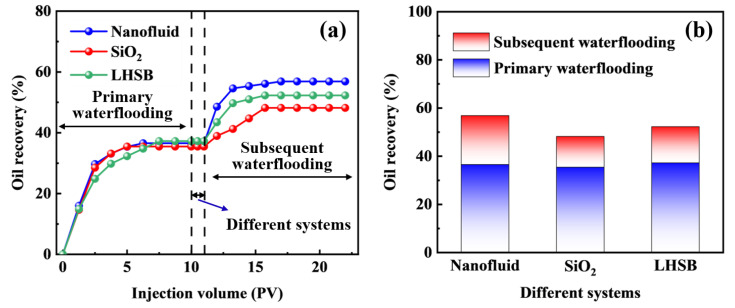
(**a**) Variation in oil recovery during oil displacement tests in different systems. (**b**) Final oil recovery for oil displacement tests in different systems.

The oil recovery curves and final oil recovery obtained with different systems are presented in Figure 6. During the primary waterflooding stage, the three systems exhibited similar oil recovery trends. After the injection of 1 PV of different systems, subsequent waterflooding further increased the oil recovery in all cases, indicating that the 0.1 wt% SiO_2_ solution, 0.1 wt% LHSB solution, and silica nanofluid were all capable of mobilizing the residual oil remaining after primary waterflooding. Among them, the silica nanofluid achieved the best oil displacement performance, with an incremental oil recovery of 20%, which was significantly higher than that of the SiO_2_ solution (13%) and the LHSB solution (15%). Compared with the individual SiO_2_ nanoparticles or LHSB, the silica nanofluid exhibited superior enhanced oil recovery performance, indicating a pronounced synergistic effect between the two components. On the one hand, LHSB effectively reduced the oil–water interfacial tension and altered the rock wettability, thereby promoting the detachment and mobilization of trapped oil. On the other hand, SiO_2_ nanoparticles exhibited strong interfacial adsorption capability and generated structural disjoining pressure while improving injectivity, which further enhanced fluid flow characteristics in porous media.

#### 2.5.2. Different Injection Volumes

**Figure 7:**
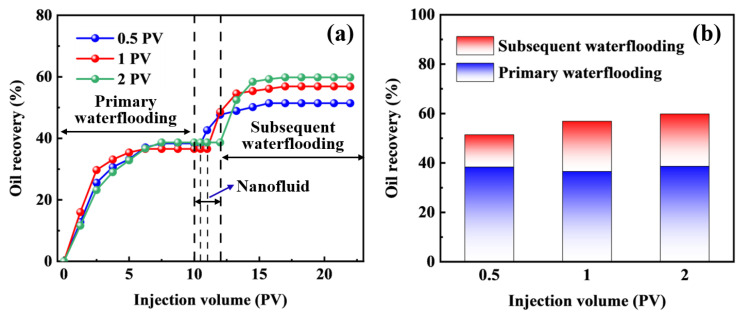
(**a**) Variation in oil recovery during oil displacement tests at different injection volumes. Each dashed line indicates a different nanofluid injection volume. (**b**) Final oil recovery for oil displacement tests at different injection volumes.

The variation in oil recovery under different silica nanofluid injection volumes is shown in Figure 7. During the primary waterflooding stage, the oil recovery curves of the three experiments exhibit similar trends, with final primary waterflooding recoveries of 38%, 37%, and 39%, respectively. After the injection of different volumes of silica nanofluid, subsequent waterflooding further improved oil recovery in all three core samples, indicating that the nanofluid can effectively mobilize the residual oil remaining in the pore space after primary waterflooding. As the nanofluid injection volume increased from 0.5 PV to 1 PV, the oil recovery during subsequent waterflooding increased significantly from 13% to 20%. This result indicates that increasing the injection volume promotes the diffusion and migration of nanofluid in porous media and enhances its contact with the rock surface, thereby improving subsequent waterflooding recovery. When the injection volume was further increased to 2 PV, the oil recovery increased only slightly to 21%, and the growth rate became much smaller, indicating that further increasing the injection volume contributes little to oil displacement performance. This is mainly because at an injection volume of 1 PV, the nanofluid has already effectively acted on the major flow channels and accessible pore space. Further increasing the injection volume is therefore unlikely to significantly expand the swept volume.

#### 2.5.3. Different Injection Slugs

**Figure 8:**
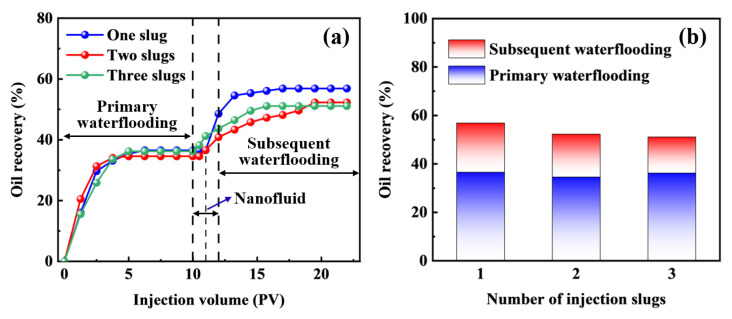
(**a**) Variation in oil recovery during oil displacement tests at different injection slugs. (**b**) Final oil recovery for oil displacement tests at different injection slugs.

The variation in oil recovery and the final oil recovery under different silica nanofluid injection slug conditions are shown in Figure 8. During the primary waterflooding stage, the oil recovery curves of the three experiments exhibit similar trends, with final primary waterflooding recoveries of 37%, 35%, and 36%, respectively. Under the condition of a constant total silica nanofluid injection volume of 1 PV, all injection slug schemes further improved oil recovery during subsequent waterflooding, indicating that silica nanofluid can effectively mobilize the residual oil remaining after primary waterflooding. However, as the number of injection slugs increased, the oil recovery during subsequent waterflooding gradually decreased from 20% to 18% and 15%, respectively, indicating that increasing the number of injection slugs is unfavorable for improving the final oil displacement performance. This is mainly because, under a constant total injection volume, an increase in slug number leads to a smaller volume for each nanofluid slug, thereby shortening the continuous action distance of the nanofluid in porous media and making it difficult to maintain sustained and sufficient interaction within major flow channels and on rock surfaces. In addition, the alternating injection of silica nanofluid and simulated formation water weakens the continuous adsorption and accumulation of nanoparticles on pore walls, thereby reducing the synergistic effects of interfacial tension reduction, wettability alteration, and flow resistance reduction. Therefore, continuous injection in a single slug is more favorable for maximizing the oil displacement potential of silica nanofluid.

#### 2.5.4. Different Shut-In Time

**Figure 9:**
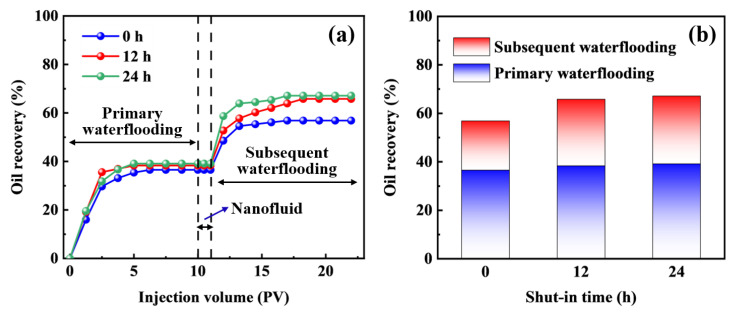
(**a**) Variation in oil recovery during oil displacement tests at different shut-in times. (**b**) Final oil recovery for oil displacement tests at different shut-in times.

The variation in oil recovery and the final oil recovery under different shut-in times are shown in Figure 9. During the primary waterflooding stage, the oil recovery curves of the three experiments exhibit similar trends, with final primary waterflooding recoveries of 37%, 38%, and 39%, respectively. After the injection of 1 PV of silica nanofluid, subsequent waterflooding further improved oil recovery under all shut-in conditions, indicating that the shut-in process is beneficial for the nanofluid to fully exert its oil displacement effect. As the shut-in time increased from 0 h to 12 h, the oil recovery during subsequent waterflooding increased significantly from 20% to 27%. However, when the shut-in time was further extended from 12 h to 24 h, the recovery increased by only 0.51%, indicating that the improvement became much less pronounced. This result suggests that further extending the shut-in time has a limited effect on enhancing oil displacement performance. This is mainly because an appropriate shut-in time promotes the further diffusion and migration of silica nanofluid in porous media and enhances the adsorption and accumulation of nanoparticles and LHSB molecules on the rock surface. As a result, the synergistic effects of interfacial tension reduction, wettability alteration, and flow resistance reduction can be more fully realized, thereby improving residual oil mobilization. However, once the shut-in time reaches a certain level, the action of the nanofluid in the reservoir gradually becomes sufficient, and further extending the shut-in time is unlikely to significantly improve interfacial regulation or expand the swept volume. Therefore, considering both oil displacement performance and operational efficiency, 12 h can be regarded as the optimal shut-in time for silica nanofluid injection.

### 2.6. Oil Displacement Mechanisms

**Figure 10:**
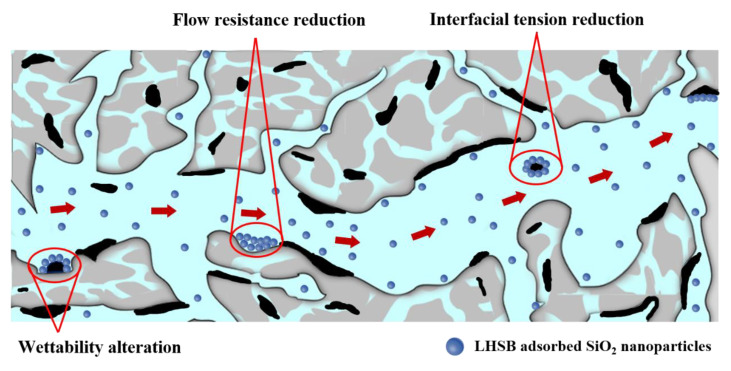
Oil displacement mechanisms of the silica nanofluid.

Combined with the interfacial properties and oil displacement performance of silica nanofluid, the potential mechanisms responsible for enhanced oil recovery can be summarized into three main aspects: interfacial tension reduction, wettability alteration, and flow resistance reduction, as illustrated in Figure 10.

#### 2.6.1. Interfacial Tension Reduction

Silica nanofluid can significantly reduce the oil–water interfacial tension, thereby weakening the restraining effect of capillary force on crude oil and lowering the threshold pressure required for crude oil to flow through pore throats. On the one hand, zwitterionic surfactant LHSB molecules can rapidly migrate to the oil–water interface and undergo directional adsorption, forming a stable adsorption film at the interface and reducing intermolecular interaction energy. On the other hand, SiO_2_ nanoparticles adsorbed with LHSB possess strong interfacial activity, which can further enhance the compactness and stability of the interfacial adsorption layer, resulting in a synergistic effect on interfacial tension reduction. The significant reduction in interfacial tension is beneficial for increasing the capillary number and promoting the mobilization and migration of trapped crude oil in pore spaces, thereby enhancing oil recovery [44,45].

#### 2.6.2. Wettability Alteration

Silica nanofluid exhibits excellent wettability alteration capability and can transform the rock surface from an oil-wet state to a water-wet state, thereby changing the distribution and flow behavior of oil and water on pore surfaces. On the one hand, nanoparticles and the adsorbed LHSB molecules on their surfaces can displace the heavy components and oil film originally adsorbed on the rock surface through competitive adsorption, thereby weakening the wettability control of crude oil on the rock surface. On the other hand, hydrophilic groups in the nanofluid can form a stable hydrophilic adsorption layer on the mineral surface, making the rock surface more easily wetted by the aqueous phase. After the wettability is altered from oil-wet to water-wet, the adhesion force between crude oil and the pore wall is significantly reduced. As a result, the displacing fluid can more easily spread along the pore surface and penetrate into fine pore throats, thereby promoting the detachment and mobilization of residual oil and ultimately enhancing oil recovery [46,47].

#### 2.6.3. Flow Resistance Reduction

Silica nanofluid exhibits a significant decompression and augmented injection effect, which can effectively reduce the flow resistance of subsequently injected simulated formation water and improve injectivity. After entering porous media, nanoparticles can adsorb onto pore-throat walls, fill microscopic rough structures, and form a nanoscale lubricating layer, thereby reducing the frictional resistance between the fluid and pore walls. Meanwhile, the reduction in interfacial tension and improvement in wettability induced by LHSB can further enhance fluid flow efficiency in complex pore networks. The synergistic effect of these two mechanisms significantly improves the seepage capacity of the displacing fluid in the reservoir. This not only facilitates deeper migration of the injected system into the formation but also increases the swept volume in porous media, thereby creating favorable conditions for enhanced oil recovery.

## 3. Materials and Methods

### 3.1. Materials

Hydrophilic silica sol (20 wt%) was supplied by Shandong Yinfeng Nano New Material Co., Ltd., Jinan, China. Lauramidopropyl hydroxy sulfobetaine (LHSB, 35 wt%), a commercially available zwitterionic surfactant, was obtained from Shandong Yousuo Chemical Technology Co., Ltd., Linyi, China. LHSB contains both quaternary ammonium and sulfonate groups in the same molecule, forming a typical zwitterionic structure. Owing to its excellent thermal stability, salinity tolerance, and interfacial activity, LHSB was selected to improve the dispersion stability of SiO_2_ nanoparticles and the interfacial properties of the prepared nanofluid. Anhydrous NaCl (99.5 wt%) and CaCl_2_ (96 wt%) were purchased from Shanghai Aladdin Reagent Co., Ltd., Shanghai, China. All chemicals were used as received without further purification. Deionized water and simulated formation water (3 wt% NaCl + 0.05 wt% CaCl_2_), formulated according to the water composition of a well in Shengli Oilfield, were prepared in the laboratory. Crude oil was also collected from Shengli Oilfield. The experimental oil phase was simulated oil prepared by mixing crude oil and kerosene at a mass ratio of 1:3, yielding a density of 0.85 g/cm^3^ and a viscosity of 1.50 mPa·s at 25 °C. Artificial sandstone cores (10 cm in length and 2.5 cm in diameter) were purchased from Beijing Anmei Kechuang Petroleum Technology Co., Ltd., Beijing, China.

### 3.2. Preparation and Characterization of the Silica Nanofluid

Zwitterionic surfactant-based silica nanofluid was prepared by dispersing 0.1 wt% silica sol and 0.1 wt% zwitterionic surfactant LHSB in deionized water. The mixture was mechanically stirred using a JJ-1 precision force multiplier electric stirrer (Changzhou Guohua Electric Appliance Co., Ltd., Changzhou, China) for 10 min, followed by ultrasonic dispersion with a JY92-IIN ultrasonic homogenizer (Shanghai Yilin Scientific Instrument Co., Ltd., Shanghai, China) for 5 min to obtain a homogeneous nanofluid. Dynamic light scattering (DLS) and zeta potential measurements of the prepared silica nanofluid and 0.1 wt% SiO_2_ solution were conducted using a NanoBrook Omni laser particle size analyzer (Brookhaven, Holtsville, NY, USA). In addition, the microstructures of the silica nanofluid and 0.1 wt% SiO_2_ solution were characterized by transmission electron microscope (TEM) using a Spectra 300 TEM (Thermo Fisher Scientiffc, Waltham, MA, USA) to evaluate the effect of LHSB addition on nanofluid stability.

### 3.3. Thermal and Salinity Stability of the Silica Nanofluid

The thermal stability of the silica nanofluid was evaluated by aging the samples at different temperatures (20, 40, 60, 80, 100, 120, and 140 °C) for 7 d, followed by measurements of particle size and zeta potential. The salinity stability was assessed by adding NaCl at different concentrations (0–7 wt%) to the silica nanofluid and measuring the corresponding particle size and zeta potential after 7 d of aging.

### 3.4. Interfacial Tension and Contact Angle Measurements

To evaluate the interfacial activity of different solutions against simulated oil, the oil–water interfacial tension was measured using the spinning drop method with a TX-500C full-range rotary interfacial tension meter (Zhongchen, Shanghai, China). The measurements were conducted at 80 °C with a rotational speed of 6000 rpm. During the test, a droplet of simulated oil was injected into a quartz sample tube pre-filled with the test solution, and the tube was then placed in the rotating chamber of the instrument. Under high centrifugal force, the oil droplet was gradually elongated and reached a stable shape. After maintaining a stable morphology for 5 min, its equilibrium image was recorded, and the oil–water interfacial tension was calculated based on the droplet length and diameter.

To investigate the wettability alteration capability of the silica nanofluid on rock surfaces, contact angle measurements were performed using a JC 2000D contact angle analyzer (Zhongchen, Shanghai, China). Cleaned and dried core slices were first vacuum-saturated with simulated oil and then aged at 80 °C for 2 weeks to establish representative oil-wet surfaces. Subsequently, a droplet of simulated formation water was placed on the treated core surface, and the initial contact angle was measured and recorded. The core slices were then immersed in the silica nanofluid for 24 h. After removal and drying of the residual fluid on the surface, another droplet of simulated formation water was deposited on the core surface, and the final contact angle was measured. The wettability alteration ability of the silica nanofluid was evaluated by comparing the contact angle before and after treatment.

### 3.5. Flow Resistance Reduction Rate Test

The flow resistance reduction performance of 0.1 wt% SiO_2_ solution, 0.1 wt% LHSB solution, and silica nanofluid was evaluated using a multifunctional flow testing system. The experimental procedure was as follows:

(1) The core samples were first cut, polished, dried, and weighed. Their porosity and permeability were then measured, and the detailed core properties are listed in Table 1. Core samples No. 1, No. 2, and No. 3 were used for the tests of 0.1 wt% SiO_2_ solution, 0.1 wt% LHSB solution, and silica nanofluid, respectively.

(2) The core samples were saturated with simulated formation water using a vacuum saturation apparatus. Both the core samples and simulated formation water were separately vacuumed for 12 h, after which the valve was opened to allow contact between the cores and the formation water under atmospheric pressure. Vacuuming was then continued for another 12 h to ensure complete saturation of the core samples. After saturation, the cores were immersed in simulated formation water for subsequent use.

(3) The two intermediate vessels in the multifunctional flow testing system were filled with simulated formation water and the test solution, respectively. The valve configuration was adjusted, and the pipeline was flushed with simulated formation water. The valve connected to the simulated formation water vessel was kept open, and the saturated core was mounted in the core holder placed inside an oven. The oven temperature was set at 80 °C, and the confining pressure applied to the core holder was maintained at 3 MPa.

(4) The ISCO pump was operated in constant-flow mode, and simulated formation water was injected into the core at a flow rate of 0.5 mL·min^−1^ for the primary waterflooding stage. The pressure differential between the inlet and outlet (at atmospheric pressure) of the core holder was continuously recorded using the built-in pressure acquisition system. Once the injection pressure differential stabilized, the ISCO pump was shut down, and the stable pressure difference *P*_1_ was recorded.

(5) The valve configuration was then adjusted to open the vessel containing the test solution while closing the simulated formation water vessel. The ISCO pump was restarted, and a predetermined volume of test solution was injected into the core at a flow rate of 0.5 mL·min^−1^. After injection, the pump was stopped.

(6) Subsequently, the valve configuration was switched back to reopen the simulated formation water vessel for the subsequent waterflooding stage. The experimental conditions were identical to those described in step (4). After the injection pressure differential reached a stable state, it was recorded as *P*_2_.

(7) The flow resistance reduction rate *S* before and after injection of different solutions was calculated according to Formula (1):

(1) $$S=\frac{P_{1}-P_{2}}{P_{1}}\times 100\%$$

where *P*_1_ is the stable pressure difference before the injection of different solutions, MPa, and *P*_2_ is the pressure differential after the injection of different solutions, MPa.

### 3.6. Oil Displacement Experiment

**Figure 11:**
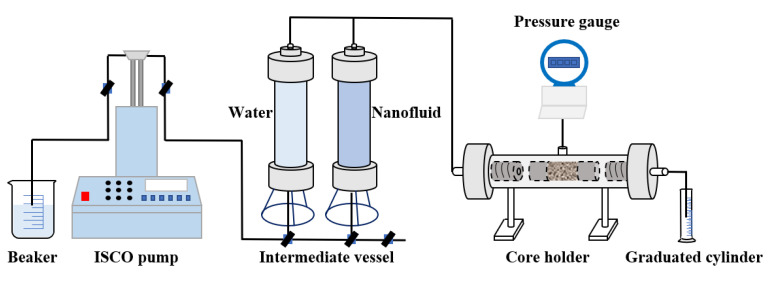
Schematic diagram of the oil displacement experimental setup.

The procedure for the oil displacement experiment was generally consistent with that of the flow resistance reduction rate evaluation, except that in step (2), the core samples were saturated with simulated oil instead of simulated formation water. During the displacement process, the cumulative oil production at the outlet was continuously recorded, and the oil recovery was subsequently calculated. A schematic diagram of the experimental setup is shown in Figure 11.

In the oil displacement experiments with different injection volumes, the injection volume of silica nanofluid was set at 0.5 PV, 1 PV, and 2 PV, with one injection slug and a shut-in time of 0 h. For the experiments with different numbers of injection slugs, the silica nanofluid was injected in one, two, and three slugs, while the total injected nanofluid volume was maintained at 1 PV. Here, different slugs refer to the alternating injection of silica nanofluid and simulated formation water. The shut-in time in these experiments was fixed at 0 h. In the oil displacement experiments with different shut-in times, the shut-in time after silica nanofluid injection was set at 0, 12, and 24 h, while the injection volume and number of injection slugs were maintained at 1 PV and one slug, respectively. The properties of the core samples used in these experiments are summarized in Table 2.

## 4. Conclusions

In this work, a silica nanofluid composed of SiO_2_ nanoparticles and the zwitterionic surfactant LHSB was successfully prepared, and its interfacial regulation characteristics and enhanced oil recovery mechanisms were systematically investigated. The introduction of LHSB effectively improved the dispersion stability of silica nanoparticles, and the prepared nanofluid maintained good stability at temperatures up to 120 °C and salinities up to 6 wt%. The silica nanofluid exhibited excellent interfacial activity and wettability alteration capability, reducing the oil–water interfacial tension to 0.57 mN/m and decreasing the static water contact angle on the rock surface from 112° to 37°. In addition, the nanofluid significantly reduced flow resistance in porous media, achieving a flow resistance reduction rate of 20%. The optimal oil displacement operating condition was determined to be 1 PV single-slug injection followed by a 12 h shut-in period, under which the oil recovery during subsequent waterflooding reached 27%. Mechanism analysis indicated that the enhanced oil recovery performance of the silica nanofluid was mainly attributed to the synergistic effects of interfacial tension reduction, wettability alteration, and flow resistance reduction.

Overall, this study provides valuable insights into the formulation design of functional nanofluids for enhanced oil recovery. However, further investigations are still required before their practical application in oil reservoirs. Future research should focus on the long-term stability of nanofluids under complex reservoir conditions, particularly in high-salinity and multivalent ion environments, as well as the transport behavior of nanoparticles in multiscale porous media. In addition, the interactions between nanoparticles, crude oil components, and rock minerals should be further clarified. The development of multifunctional nanofluids through surface functionalization, together with molecular simulations, microfluidic visualization, and field-scale validation, will contribute to a deeper understanding of the mechanisms of nanofluid-enhanced oil recovery. These efforts are expected to provide a more comprehensive theoretical foundation and technical support for the efficient development of low-permeability reservoirs.

## Figures and Tables

**Table 1:** Specific core parameters of flow resistance reduction rate test.

Number	Length/cm	Diameter/cm	Permeability/mD	Porosity/%
1	4.84	2.52	2.21	18.87
2	4.89	2.53	2.21	18.27
3	4.96	2.53	2.16	17.78

**Table 2:** Specific core parameters of oil displacement experiment.

Number	Length /cm	Diameter /cm	Permeability /mD	Porosity/%	Injection Volume/PV	Injection Slug	Shut-In Time/h
4	4.86	2.52	2.14	18.06	0.5	1	0
5	4.78	2.52	2.05	18.09	1	1	0
6	4.92	2.52	2.17	18.14	2	1	0
7	4.93	2.52	2.15	17.85	1	2	0
8	4.75	2.52	2.15	17.62	1	3	0
9	4.89	2.52	2.18	18.23	1	1	12
10	4.90	2.52	2.20	18.71	1	1	24

## Data Availability

The original contributions presented in this study are included in the article. Further inquiries can be directed to the corresponding author.
